# Lack of association between modifiable exposures and glioma risk: a Mendelian randomization analysis

**DOI:** 10.1093/neuonc/noz209

**Published:** 2019-10-30

**Authors:** Charlie N Saunders, Alex J Cornish, Ben Kinnersley, Philip J Law, Elizabeth B Claus, Dora Il’yasova, Joellen Schildkraut, Jill S Barnholtz-Sloan, Sara H Olson, Jonine L Bernstein, Rose K Lai, Stephen Chanock, Preetha Rajaraman, Christoffer Johansen, Robert B Jenkins, Beatrice S Melin, Margaret R Wrensch, Marc Sanson, Melissa L Bondy, Richard S Houlston

**Affiliations:** 1 Division of Genetics and Epidemiology, The Institute of Cancer Research, London, UK; 2 Department of Population and Quantitative Health Sciences and the Cleveland Center for Health Outcomes Research, Case Western Reserve University School of Medicine, Cleveland, Ohio, USA; 3 Section of Epidemiology and Population Sciences, Department of Medicine, Dan L. Duncan Comprehensive Cancer Center, Baylor College of Medicine, Houston, Texas, USA; 4 School of Public Health, Yale University, New Haven, Connecticut, USA; 5 Department of Neurosurgery, Brigham and Women’s Hospital, Boston, Massachusetts, USA; 6 Department of Epidemiology and Biostatistics, School of Public Health, Georgia State University, Atlanta, Georgia, USA; 7 Duke Cancer Institute, Duke University Medical Center, Durham, North Carolina, USA; 8 Cancer Control and Prevention Program, Department of Community and Family Medicine, Duke University Medical Center, Durham, North Carolina, USA; 9 Department of Epidemiology and Biostatistics, Memorial Sloan Kettering Cancer Center, New York, New York, USA; 10 Departments of Neurology and Preventive Medicine, Keck School of Medicine, University of Southern California, Los Angeles, California, USA; 11 Division of Cancer Epidemiology and Genetics, National Cancer Institute, Bethesda, Maryland, USA; 12 Danish Cancer Society Research Center, Survivorship, Danish Cancer Society, Copenhagen, Denmark; 13 Oncology Clinic, Finsen Centre, Rigshospitalet, University of Copenhagen, Copenhagen, Denmark; 14 Department of Laboratory Medicine and Pathology, Mayo Clinic Comprehensive Cancer Center, Mayo Clinic, Rochester, Minnesota, USA; 15 Department of Radiation Sciences, Umeå University, Umeå, Sweden; 16 Department of Neurological Surgery, School of Medicine, University of California San Francisco (UCSF), San Francisco, California, USA; 17 Institute of Human Genetics, University of California San Francisco, San Francisco, California, USA; 18 Sorbonne University, National Center for Scientific Research, National Institute of Health and Medical Research (INSERM), Brain and Spinal Cord Institute, Paris, France; 19 Department of Neurology Mazarin 2, Pitié-Salpêtrière Hospital Group, Paris, France; 20 Division of Molecular Pathology, The Institute of Cancer Research, London, UK

**Keywords:** cancer, glioma, Mendelian randomization, risk

## Abstract

**Background:**

The etiological basis of glioma is poorly understood. We have used genetic markers in a Mendelian randomization (MR) framework to examine if lifestyle, cardiometabolic, and inflammatory factors influence the risk of glioma. This methodology reduces bias from confounding and is not affected by reverse causation.

**Methods:**

We identified genetic instruments for 37 potentially modifiable risk factors and evaluated their association with glioma risk using data from a genome-wide association study of 12 488 glioma patients and 18 169 controls. We used the estimated odds ratio of glioma associated with each of the genetically defined traits to infer evidence for a causal relationship with the following exposures:

Lifestyle and dietary factors—height, plasma insulin-like growth factor 1, blood carnitine, blood methionine, blood selenium, blood zinc, circulating adiponectin, circulating carotenoids, iron status, serum calcium, vitamins (A1, B12, B6, E, and 25-hydroxyvitamin D), fatty acid levels (monounsaturated, omega-3, and omega-6) and circulating fetuin-A;

Cardiometabolic factors—birth weight, high density lipoprotein cholesterol, low density lipoprotein cholesterol, total cholesterol, total triglycerides, basal metabolic rate, body fat percentage, body mass index, fasting glucose, fasting proinsulin, glycated hemoglobin levels, diastolic and systolic blood pressure, waist circumference, waist-to-hip ratio; and

Inflammatory factors— C-reactive protein, plasma interleukin-6 receptor subunit alpha and serum immunoglobulin E.

**Results:**

After correction for the testing of multiple potential risk factors and excluding associations driven by one single nucleotide polymorphism, no significant association with glioma risk was observed (ie, *P*_*Corrected*_ > 0.05).

**Conclusions:**

This study did not provide evidence supporting any of the 37 factors examined as having a significant influence on glioma risk.

**Key Points**

- Environmental modifiable risk factors effecting glioma risk are poorly understood.

- Our Mendelian randomization study investigated 37 potential modifiable risk factors.

- None of the 37 factors were observed to significantly influence glioma risk.

**Importance of the Study**

In this study we attempt to elucidate modifiable risk factors of glioma, the etiological basis of which is poorly understood. We used genetic markers in an MR framework to examine whether the risk of glioma is influenced by one of 37 lifestyle, cardiometabolic, and inflammatory factors. The MR methodology reduces bias from confounding and is not affected by reverse causation, an improvement over the traditional observational studies that have previously been conducted. Additionally, we leverage the largest glioma genome-wide association study dataset published to date, giving our analysis more power compared with other studies of its type. This improved, unbiased, and well-powered assessment of potential glioma risk factors provides invaluable information to the field.

Gliomas account for around 80% of malignant primary brain tumors in adults.^1^ Gliomas are heterogeneous, and different tumor subtypes can be broadly classified into glioblastoma (GBM) and lower-grade glioma (non-GBM). Gliomas are typically associated with a poor prognosis, irrespective of clinical care, with the most common glioma subtype (GBM) being associated with a median overall survival of only 12 months.^2^

While glioma subtypes have distinct molecular profiles presumably resulting from different etiological pathways, no environmental exposures have consistently been linked to risk, except for ionizing radiation, which accounts for only a very small number of cases.^2,3^ However, the near threefold higher incidence in northern Europe (6.59 cases per 100 000) than in prosperous Southeast Asia (2.55 cases per 100 000) raises the possibility that lifestyle factors influence glioma risk.^2,4^ Over the last 30 years, observational epidemiological studies have sought to establish associations between a variety of lifestyle factors and risk of developing glioma. Most studies have focused on factors previously shown to influence risk of other cancers, such as diet. Results from these observational epidemiological studies have so far either been inconsistent, null, or not independently validated; for example, the conflicting evidence for possible associations with dietary factors and obesity.^5–11^ In contrast to other cancer types, published studies have shown an inverse relationship with both diabetes and hyperglycemia for glioma.^12^ Studies of a possible relationship between metabolic syndrome traits (triglyceride [TG] and cholesterol levels, body fat and blood pressure) have produced similarly mixed results.^8,13,14^

Associations seen in conventional observational studies may not be causal, instead arising as a consequence of methodological biases inherent in the study design. Biases include selection bias in controls, recall bias, reverse causation, or confounding from unmeasured effects.^15^ Furthermore, the high frequency of exposure ascertainment by proxy in studies of glioma represents an additional source of bias.^16^

Mendelian randomization (MR) is an analytical approach, whereby germline genetic variants are used as proxies, or instrumental variables (IVs), for putative risk factors.^17^ Since genetic variants are randomly assigned at conception, they are not influenced by reverse causation. In the absence of pleiotropy (ie, genetic variants being associated with the disease through alternative pathways) and population stratification, they can provide unconfounded estimates of disease risk (Fig. 1).^17^ MR can therefore mitigate many of the limitations of conventional observational studies and is increasingly being used to estimate the impact of an intervention on disease risk.

**Fig. 1:**
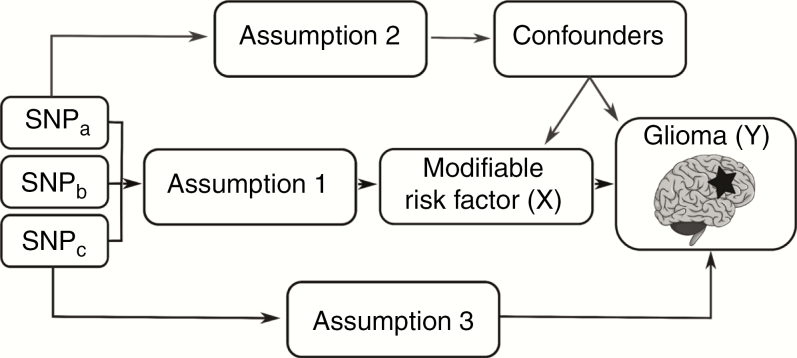
Principles of Mendelian randomization (MR) and the assumptions required to obtain an unbiased causal effect estimate. The three assumptions are: (1) genetic variants used as instrumental variables are only associated with the modifiable risk factor (X); (2) genetic variants only influence the risk of developing glioma (Y) through the modifiable risk factor (X); (3) genetic variants are not associated with any measured or unmeasured confounders.

We have recently used MR to examine possible links between glioma with 25-hydroxyvitamin D levels, a common proxy for vitamin D levels, obesity, and atopy-related traits.^18–20^ Here we have extended our analysis to examine the relationship of 37 potentially modifiable factors with glioma risk, using a two-sample MR framework. Genetic variants associated with these 37 factors were identified from the largest genome-wide association study (GWAS) conducted to date or meta-analysis of each trait. We then evaluated the association of these variants with glioma in a large GWAS comprising 12 488 glioma cases and 18 169 control subjects.^21^

## Methods

Two-sample MR was conducted using data from a GWAS of glioma published by Melin et al.^21^ Ethical approval was not sought because these data came from summary statistics and no individual-level data were used.

### Potentially Modifiable Risk Factors

The aim of our study was to provide an insight into possible associations between modifiable factors which might influence the risk of glioma development. These factors were chosen on the basis of having been the subject of a previous investigation or having a role in development of a common cancer (Supplementary Table 1). Specifically, we considered 19 lifestyle and dietary factors—height, plasma insulin-like growth factor 1 (IGF-1), blood carnitine, blood methionine, blood selenium, blood zinc, circulating adiponectin, circulating carotenoids, iron status, serum calcium, vitamins (A1, B12, B6, E, and 25-hydroxyvitamin D), fatty acid levels (mono-unsaturated, omega-3, and omega-6) and circulating fetuin-A. Additionally, 15 cardiometabolic factors were considered—birth weight, high density lipoprotein (HDL) cholesterol, low density lipoprotein (LDL) cholesterol, total cholesterol, total TG, basal metabolic rate, body fat percentage, body mass index, diastolic blood pressure, fasting glucose, fasting proinsulin, glycated hemoglobin (HbA1C) levels, systolic blood pressure, waist circumference, waist-to-hip ratio. Lastly, 3 inflammatory factors—C-reactive protein (CRP), interleukin (IL)-6, and serum immunoglobulin (Ig)E—were used as general markers of systemic inflammation and were included in the hypothesis driven analysis (Supplementary Table 2).

### Genetic Instruments for Putative Risk Factors

The genetic instruments (ie, single nucleotide polymorphisms [SNPs]) to be used as IVs were identified from recent meta-analyses or the largest GWAS published to date (Supplementary Table 3). For each SNP, the chromosome position was recovered, the effect estimate expressed in standard deviations (SDs) of the trait per allele along with the corresponding standard errors (SEs). We considered only continuous traits, as analysis of binary traits (such as disease status) with binary outcomes in 2-sample MR frameworks can result in inaccurate causal estimates.^18,19^ The analysis was restricted to SNPs associated at genome-wide significance (ie, *P* ≤ 5 × 10^−8^) in individuals of European ancestry, to satisfy the MR assumption that genetic variants are associated with the modifiable risk factor.^22^ To avoid collinearity between SNPs for each trait, correlated SNPs were excluded using the MR-Base database (linkage disequilibrium threshold, r^2^ ≥ 0.01) within each trait, with SNPs with the strongest effect size retained.^23^ These SNPs and their associated data are detailed in Supplementary Table 3. The process to generate SNPs used as IVs is summarized in Supplementary Fig. 1.

### Glioma Genotyping Data

The association of each genetic instrument with glioma risk was examined using summary effect estimates and corresponding SEs from a recent meta-analysis of 8 glioma GWAS.^21^ After imputation, this meta-analysis related >10 million genetic variants to glioma in 12 488 cases (6183 GBM and 5820 non-GBM) and 18 169 controls of European descent (Supplementary Table 4).

### Statistical Analysis

The MR methodology assumes that genetic variants used as instruments for a risk factor are associated with only the risk factor and not with any confounders or another causal pathway (Fig. 1). Furthermore, to estimate the size of the causal effect with precision, associations must be linear and not affected by statistical interactions.^24^ The causal effects for each SNP were first estimated using the Wald ratio (Supplementary Table 5). Where multiple SNPs were available as instruments for the trait, causal effects were estimated using an inverse variance weighted fixed-effects (IVW-FE), maximum likelihood estimation (MLE), weighted median estimator (WME), and weighted mode-based estimator (WMBE) methodologies.^17,25,26^ We compared the calculated odds ratios and *P*-values from the 4 methods to assess the stability and validity of associations. Leave-one-out analysis was used to investigate whether a particular association was driven solely by a single SNP (Supplementary Table 6).^27^ The MR-Egger regression approach was used to evaluate the extent to which directional pleiotropy may affect the causal estimates.^28^ Results were reported as odds ratios (OR_SD_) with 95% confidence intervals (CIs) per genetically predicted SD unit increase in each putative risk factor. To address multiple testing, a Bonferroni-corrected *P*-value of 1.35×10^−3^ (ie, 0.05/37 putative risk factors) was considered significant, with a 1.35×10^−3^ < *P*-value < 0.05 being considered suggestive of an association. The power of MR to demonstrate a causal effect depends in part on the proportion of variance in the risk factor explained by the genetic variants used as instruments, and we therefore estimated study power for each risk factor *a priori* (Supplementary Table 2). Statistical analyses were undertaken using RStudio version 3.4.0^29^ and MR-Base.^23^ Figures were produced using Inkscape version 0.92.^30^

## Results

**Fig. 2:**
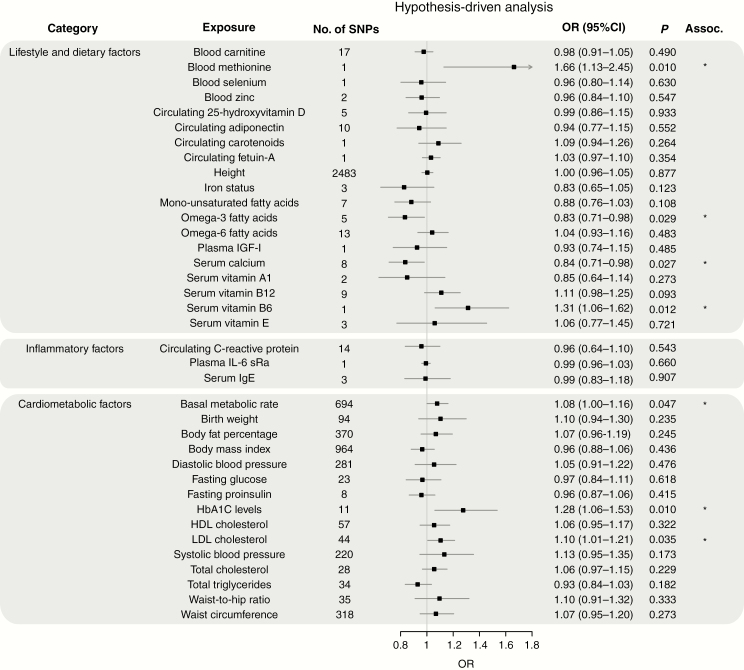
Odds ratios for associations (Assoc.) between genetically predicted risk factors and glioma. Results reported as odds ratios (OR_SD_) and 95% confidence intervals (CIs) per genetically predicted standard deviation (SD) unit increase in the risk factor. A fixed-effects inverse variance weighted (IVW-FE) method was used to summarize Wald ratio estimates from individual SNPs. *P-values suggestive of an association (range: 0.05–1.35 × 10^−3^); **significant *P*-values (<1.35 × 10^−3^).

**Fig. 3.**
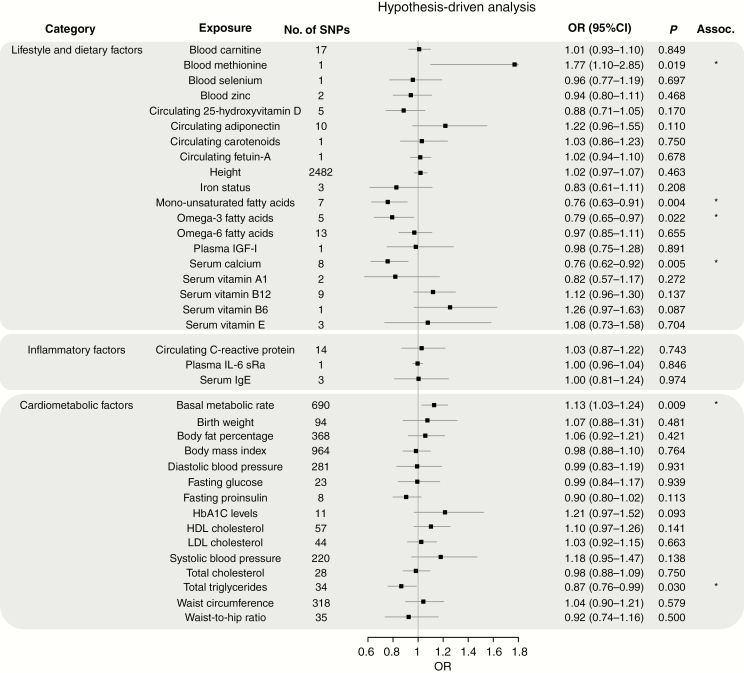
Odds ratios for associations (Assoc.) between genetically predicted risk factors and GBM. Results reported as odds ratios (OR_SD_) and 95% CIs per genetically predicted SD unit increase in the risk factor. A fixed-effects inverse variance weighted (IVW-FE) method was used to summarize Wald ratio estimates from individual SNPs. *P-values suggestive of an association (range: 0.05–1.35 × 10^−3^); **significant *P*-values (<1.35 × 10^−3^).

**Fig. 4:**
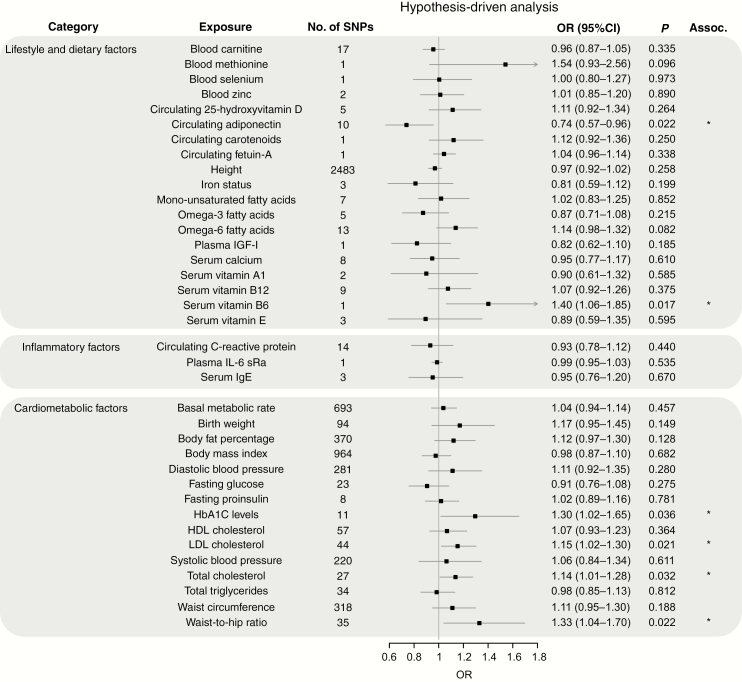
Odds ratios for associations (Assoc.) between genetically predicted risk factors and non-GBM. Results reported as odds ratios (OR_SD_) and 95% CIs per genetically predicted SD unit increase in the risk factor. A fixed-effects inverse variance weighted (IVW-FE) method was used to summarize Wald ratio estimates from individual SNPs. *P-values suggestive of an association (range: 0.05–1.35 × 10^−3^); **significant *P*-values (<1.35 × 10^−3^).

Figures 2–4 show the association between each of the 37 traits and risk of all glioma, GBM and non-GBM tumors, respectively, using the Wald ratio and IVW-FE methodologies.

### Dietary Factors and Lifestyle

There was suggestive evidence of an association between genetically predicted higher basal metabolic rate with increased risk of all glioma (OR_SD_ = 1.08, 95% CI: 1.00–1.16, *P* = 0.047) and GBM (OR_SD_ = 1.13, 95% CI: 1.03–1.24, *P* = 0.009). However, leave-one-out analysis showed the association was driven by the SNP rs78378222 at 17p13.3 (Supplementary Table 6), a known glioma risk SNP.^31^ With exclusion of rs78378222, no association was observed in glioma (OR_SD_ = 1.04, 95% CI: 0.96–1.12, *P* = 0.319) and GBM (OR_SD_ = 1.09, 95% CI: 0.99–1.19, *P* = 0.072). Likewise, the apparent association between genetically predicted raised serum calcium levels and a lower risk of glioma (OR_SD_ = 0.84, 95% CI: 0.71–0.98, *P* = 0.027) and GBM (OR_SD_ = 0.76, 95% CI: 0.62–0.92, *P* = 0.005) was reliant on SNP rs780094 (Supplementary Table 6). With exclusion of rs780094 the association was non-significant with glioma (OR_SD_ = 0.92, 95% CI: 0.69–1.23, *P* = 0.574) and GBM (OR_SD_ = 0.86, 95% CI: 0.60–1.22, *P* = 0.391). There was suggestive evidence for genetically predicted higher serum vitamin B6 levels being associated with lower risk of all glioma (OR_SD_ = 1.31, 95% CI: 1.06–1.62, *P =* 0.012) and non-GBM (OR_SD_ = 1.40, 95% CI: 1.06–1.85, *P =* 0.017); however, the observation was again reliant on a single SNP (rs4654748). Similarly, the suggestive association between genetically predicted higher blood methionine levels and risk of glioma (OR_SD_ = 1.66, 95% CI: 1.13–2.45, *P =* 0.010) and GBM (OR_SD_ = 1.77, 95% CI: 1.10–2.85, *P =* 0.019) was reliant on a single SNP (rs320485).

The fatty acid (FA) metabolic pathway is complex, with SNPs influencing the metabolism of one FA often being associated with circulating concentrations of multiple FAs. To limit bias introduced by vertical and horizontal pleiotropy, we restricted our analysis to classes of FAs, such as omega-3 and omega-6 polyunsaturated FAs (PUFAs) and monounsaturated FAs (MUFAs), rather than individual fatty acids. In this restricted analysis, there was a suggestive association between genetically predicted levels of MUFA with reduced risk of GBM (OR_SD_ = 0.76, 95% CI: 0.63–0.91, *P =* 0.004) and between omega-3 PUFA fatty acid levels with reduced risk of glioma (OR_SD_ = 0.83, 95% CI: 0.71–0.98, *P =* 0.029) and GBM (OR_SD_ = 0.79, 95% CI: 0.65–0.97, *P =* 0.022). Leave-one-out analysis, however, revealed that all associations were driven by the same glucokinase receptor protein (*GCKR*) SNP (rs1260326, Supplementary Table 6). With the exclusion of this SNP, no association remained significant.

Circulating adiponectin level was the only exposure with an observed association with non-GBM risk only (OR_SD_ = 0.74, 95% CI: 0.57–0.96, *P =* 0.022). However, once again leave-one-out analysis showed this association was reliant on rs6810075; upon its removal the association did not remain significant (OR_SD_ = 0.75, 95% CI: 0.55–1.04, *P =* 0.086).

Genetically predicted circulating levels of carnitine/selenium/zinc/25-hydroxyvitamin-D/carotenoids/fetuin-A/plasma IGF-1/vitamins (A1, B12, and E), height, iron status, and circulating levels of monounsaturated/omega-3/omega-6 fatty acids showed no evidence for association with risk of all glioma, GBM or non-GBM (Fig. 2–4).

### Cardiometabolic and Inflammatory Factors

Genetically predicted higher levels of LDL cholesterol showed suggestive evidence of an association with increased risk of glioma (OR_SD_ = 1.10, 95% CI: 1.01–1.21, *P =* 0.035) and non-GBM (OR_SD_ = 1.15, 95% CI: 1.02–1.30, *P =* 0.021). This finding contrasts with our earlier work which found no evidence for an association; however, the previous analysis was based on fewer SNPs (26 vs 44).^18^ Genetically predicted plasma total TG also showed a suggestive association with risk of GBM (OR_SD_ = 0.87, 95% CI: 0.76–0.99, *P* = 0.030). Leave-one-out analysis showed that both LDL and TG associations were unstable. The LDL association being reliant on rs2131925 (Supplementary Table 6), with exclusion of rs2131925 removing any association–glioma (OR_SD_ = 1.09, 95% CI: 0.99–1.19, *P =* 0.085) and non-GBM risk (OR_SD_ = 1.12, 95% CI: 0.98–1.27, *P =* 0.090). The TG association was reliant on the *GCKR* SNP (rs1260326, Supplementary Table 6), with exclusion of rs1260326 also leading to the loss of association–GBM OR_SD_ = 0.93, 95% CI: 0.81–1.08, *P =* 0.360).

Genetically predicted higher HbA1C levels were also associated with increased glioma risk (OR_SD_ = 1.28, 95% CI: 1.06–1.53, *P =* 0.010) and non-GBM (OR_SD_ = 1.30, 95% CI: 1.02–1.65, *P =* 0.036). However, this association was also reliant on a single SNP (Supplementary Table 6), with the exclusion of rs16926246 resulting in loss of any association–glioma (OR_SD_ = 1.16, 95% CI: 0.93–1.45, *P =* 0.177) and non-GBM risk (OR_SD_ = 1.15, 95% CI: 0.86–1.54, *P =* 0.346).

Genetically predicted total cholesterol was associated with non-GBM risk only (OR_SD_ = 1.14, 95% CI: 1.01–1.28, *P =* 0.032). However, this association was also reliant on a single SNP (Supplementary Table 6b), with the exclusion of rs7412 resulting in loss of any association (OR_SD_ = 1.11, 95% CI: 0.97–1.27, *P =* 0.116).

Waist-to-hip ratio was associated with non-GBM risk only (OR_SD_ = 1.33, 95% CI: 1.04–1.70, *P =* 0.022). Leave-one-out analysis of this exposure revealed that removal of any one of 3 SNPs (rs10195252, rs1936805, or rs2820443) reduced the *P*-value to just above the 0.05 threshold for a suggestion of association (*P* = 0.059, 0.055, and 0.053, respectively). This was less than the drastic reduction seen with the other exposures.

Genetically predicted plasma levels of IL-6 receptor subunit alpha (sRa)/CRP/HDL and serum IgE, birth weight, body fat percentage, body mass index, diastolic and systolic blood pressure, fasting glucose and proinsulin levels, and waist circumference showed no evidence for association with risk of all glioma, GBM or non-GBM (Fig. 2–4).

## Discussion

Despite much research, the etiological basis of glioma has remained elusive. To gain insight into possible causal relationships, we have used an MR-based framework to investigate a range of potentially modifiable risk factors. Many of the factors and traits have either been the subject of previous conventional observational epidemiological studies with varying degrees of support or are established risk factors for multiple common cancers consistent with them having a generic effect on tumor development.

A major advantage of the MR approach to establish causal links is the avoidance of biases that can influence conventional observational epidemiological studies. A challenge in its implementation is exclusion of pleiotropy, where one SNP is seen to effect two seemingly unrelated phenotypic traits, or an alternative direct causal pathway being the cause of an association.^32^ The IVW methodology only produces estimates of causal relationship when all genetic variants are valid instruments. To address such a shortcoming, and assess the robustness of estimates, as well as implementing IVW we also made use of WME and WMBE methods, which can provide unbiased causal effect estimates even when many genetic variants are invalid instruments.^25,26^ While not a case of direct pleiotropy, our analysis did show one example of a pleiotropic locus influencing both GCKR SNP (rs1260326) and serum calcium (rs780094). However, both of these exposures were discounted after leave-one-out analysis showed them both to be reliant on these single SNPs, before the need to investigate the association using WME or WMBE methodologies.

None of the 37 potential risk factors we evaluated showed a significant association with glioma risk after adjusting for multiple testing (ie, *P* < 1.35 × 10^−3^) although 9 showed suggestive evidence (ie, *P* < 0.05). OR_SD_ and *P-*values estimated using IVW and MLE methods showed strong agreement with respect to the 9 suggestively associated factors. Only two exposures showed consistent effect estimates across all 4 analytic methodologies (including WME and WMBE), highlighting the instability of any suggested associations identified by one method alone (Supplementary Table 5). These consistent exposures were HbA1C levels (GBM and non-GBM only) and serum calcium (GBM only); however, leave-one-out analysis showed that these associations were reliant on single SNPs, thereby calling into question the validity of associations.

We cannot exclude the possibility that some of our findings have been affected by weak instrument bias, despite all factors having high F-statistics (>10) (Supplementary Table 2). For all glioma, we had sufficient power to demonstrate a causal relationship (ie, >80%) and detect OR_SD_ of 1.33 for all but 5 risk factors. However, we only had >80% power to detect an OR_SD_ of 1.10 for 5 traits. Moreover, our power to demonstrate subtype-specific associations was even more restricted (Supplementary Table 2a and 2b, respectively). Hence, we cannot exclude the possibility that some of the traits examined may have very modest effects on glioma risk.

Accepting these caveats, in conclusion our analysis provides no convincing evidence to support any of the 37 potentially modifiable factors we examined having a significant association with glioma risk.

## Supplementary Material

noz209_suppl_Supplementary_Figure_S1

noz209_suppl_Supplementary_Tables_ST2-ST8

noz209_suppl_Supplementary_Tables_ST2a-ST8a

noz209_suppl_Supplementary_Tables_ST2b-ST8b
