# Supplementary figures and images for: Lack of association between modifiable exposures and glioma risk: a Mendelian randomization analysis
Source: Neuro Oncol. 2019 Oct 30;22(2):207–15. doi: 10.1093/neuonc/noz209 (PMC7442418; doi:10.1093/neuonc/noz209)

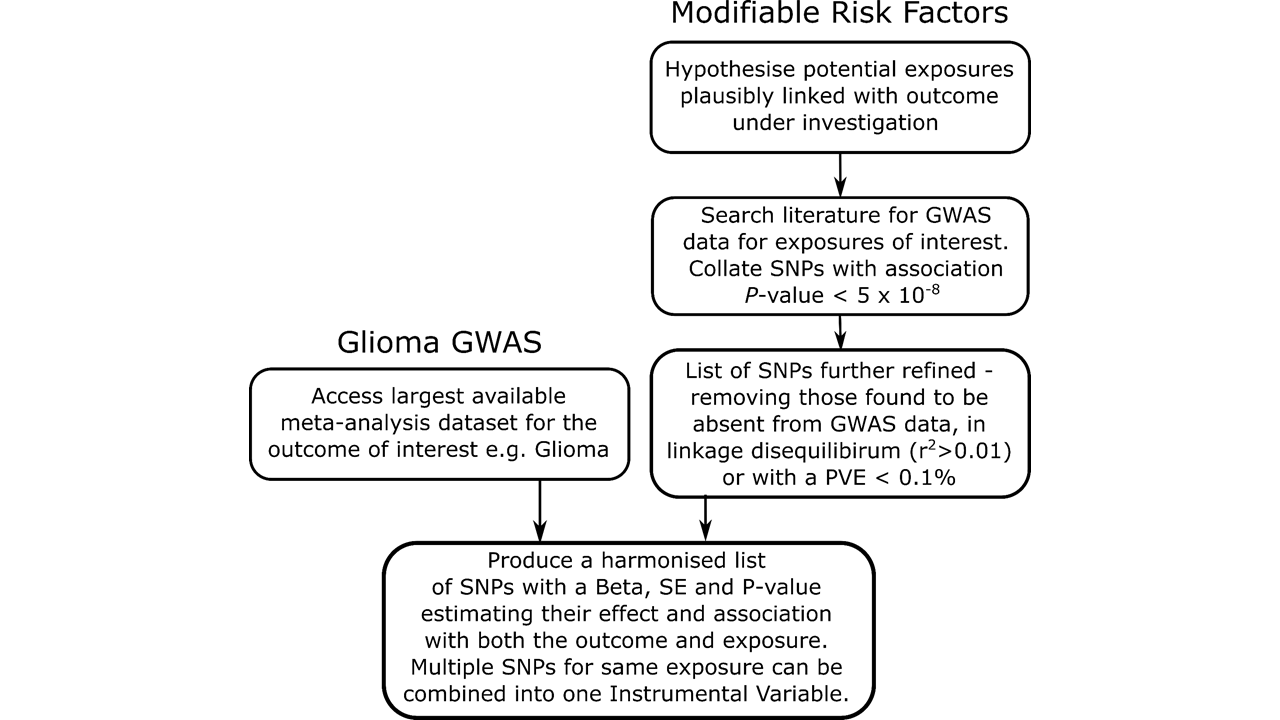

Supplement: noz209_suppl_Supplementary_Figure_S1 [file noz209_suppl_supplementary_figure_s1.png]
